# An Account of Diseases in an Eastern District of London, from February 20, to March 20, 1808

**Published:** 1808-04

**Authors:** 


*An Account of Diseases in an Eastern District of London, from February 20, to March 20, 1808.*

| | |
|---|---|
| **ACUTE DISEASES.** | |
| Pneumonia | 4 |
| Peripneumonia Notha | 11 |
| Abortus | 1 |
| Ophthalmia | 9 |
| Rubeola | 5 |
| Rheumatismus Acutus | 5 |
| **CHRONIC DISEASES.** | |
| Tussis | 15 |
| Tussis cum Dyspnœa | 23 |
| Dyspnœa | 9 |
| Phthisis Pulmonalis | 3 |
| Pleurodyne | 4 |
| Hæmoptysis | 3 |
| Hepatitis Chronica | 1 |
| Cephalalgia | 4 |
| Vertigo | 3 |
| Paralysis | 2 |
| Chorea | 1 |
| Dyspepsia | 7 |
| Hypochondriasis | 2 |
| Dysuria | 5 |
| Amenorrhœa | 3 |
| Fluor Albus | 7 |
| Rheumatismus Chronicus | 17 |
| **PUERPERAL DISEASES.** | |
| Menorrhagia Lochialis | 7 |
| Dysuria | 3 |
| Hæmorrhois | 5 |
| Mastodynia | 4 |
| **INFANTILE DISEASES.** | |
| Febris Mesenterica | 2 |
| Aphthæ | 4 |
| Vermes | 5 |

The Report which is so frequently made at this season of the year must again be repeated; and, when it is recollected to what a long period the easterly and north-easterly winds have been protracted, and with how much severity of cold they were attended, it will not appear surprising that so large a proportion of the list is occupied by diseases of the chest, both of the acute and chronic kind. Many of the patients referred to, had laboured under one or other of the thoracic affections for several years. Every winter brought with it, cough, dyspnœa, pain in the side, or some other complaint of the chest: and every attack left the patient more subject to a return of the disease, and at the same time rendered him less capable of struggling with it. In several instances the disease has proceeded to a fatal termination; nor can it be wondered at, that a complaint which has its seat in organs of the first importance to life, and which commonly occurs to persons at an advanced age, should baffle all medical skill.

CRITICAL

